# Sensitization of ovarian cancer cells to cisplatin by genistein: the role of NF-kappaB

**DOI:** 10.1186/1757-2215-1-9

**Published:** 2008-11-24

**Authors:** Leigh A Solomon, Shadan Ali, Sanjeev Banerjee, Adnan R Munkarah, Robert T Morris, Fazlul H Sarkar

**Affiliations:** 1 Division of Gynecologic Oncology, Karmanos Cancer Center, Wayne State University, Detroit, Michigan, USA; 2 Department of Internal Medicine, Karmanos Cancer Center, Wayne State University, Detroit, Michigan, USA; 3 Department of Pathology, Karmanos Cancer Center, Wayne State University, Detroit, Michigan, USA; 4 Division of Gynecologic Oncology, Henry Ford Hospital, Detroit, Michigan, USA

## Abstract

**Background:**

Platinum-resistance (PR) continues to be a major problem in the management of epithelial ovarian cancer (EOC). Response to various chemotherapeutic agents is poor in patients deemed PR. Genistein, a soy isoflavone has been shown to enhance the effect of chemotherapy in prostate and pancreatic cancer cells *in vitro *and *in vivo *by reversing chemo-resistance phenotype. The goal of this study was to investigate the effects of combination therapy with genistein and cisplatin as well as other cytotoxic conventional chemotherapeutic agents in platinum-sensitive (PS) and resistant EOC cells.

**Methods:**

The PS human ovarian cancer cell line A2780 and its PR clone C200 cells were pretreated with genistein, followed by the combination of genistein and either cisplatin, taxotere or gemcitabine. Cell survival and apoptosis was assessed by MTT and histone-DNA ELISA. Electrophoretic mobility shift assay (EMSA) was used to evaluate NF-κB DNA binding activity. Western blot analysis was performed with antibodies to Bcl-2, Bcl-xL, survivin, c-IAP and PARP.

**Results:**

Reduction in cell viability, and corresponding induction of apoptosis was observed with genistein pretreatment followed by combination treatment with each of the drugs in both cell lines. The PS cell line was pretreated for 24 hours; in contrast, the PR cell line required 48 hours pretreatment to achieve a response. The anti-apoptotic genes c-IAP1, Bcl-2, Bcl-xL, survivin and NF-κB DNA binding activity were all found to be down-regulated in the combination groups.

**Conclusion:**

This study convincingly demonstrated that the current strategy can be translated in a pre-clinical animal model, and thus it should stimulate future clinical trial for the treatment of drug-resistant ovarian cancer.

## Background

There will be an estimated 15,520 deaths from ovarian carcinoma and 21,650 new cases diagnosed in 2008 [1]. Unfortunately, at the time of diagnosis the majority of patients will have disseminated disease. Resistance to platinum-containing regimens and tumor heterogeneity confers a poor prognosis in patients with epithelial ovarian cancer. Platinum-resistance is a complex issue and is currently believed to be associated with an unstable phenotype of ovarian cancer cells that are believed to be altered by tumor microenvironment and exposure to other drugs [2,3]. Acquisition of chemo-resistance is one of the major limitations for the use of platinum complexes in cancer chemotherapy. Proposed mechanisms of cellular resistance include decreased cellular uptake of the toxic drug, increased cell efflux of the drug, improved cell DNA damage repair and the prevention of DNA cross-linking. These may be intrinsic properties of some cancer cells or acquired mechanisms due to exposure to chemotherapeutic agents.

Over the past few years it has been shown that a small portion of cancer cells known as "cancer stem cells" or "cancer stem-like cells" are responsible for the antagonism of the disease, resistance to therapy, self-renewal and unlimited proliferation in several cancers, including ovarian cancer [4-7]. Moreover, mutations may be one of the major factors contributing to the origin of ovarian cancer stem cells. Emerging evidence suggests that ovarian cancer stem cells are relatively resistant to conventional cytotoxic chemotherapeutic agents [8]. These therapies often cause severe toxicity because of their general effects on all rapidly dividing cells. It is important that we use targeted agents that discriminate between cancer stem cells and normal stem cells. One such agent which has been studied in our laboratory and by others is "genistein", a naturally occurring isoflavone present in soybeans has proven to have anti-tumor activity with minimal or no toxicity to nonmalignant human cells [9,10]. Moreover, the incidence of ovarian cancer is approximately 10–50% lower in Asian countries compared to the United States [11], which could be associated with dietary factors. Asian women who migrate to the United States and their descendents seem to maintain the decreased risk [11]. In a case control study in Southeast China, Zhang, et al, found the odds ratio of developing ovarian cancer with a diet high in genistein to be half that of controls [12], and suggest that soy isoflavone may contribute to reduced cancer risk in Asian population [12].

Studies of various cancer cell lines, in our laboratory and others, have shown that treatment with the isoflavonoid genistein can inhibit cell proliferation. In the breast cancer cell line MDA-MB-231, treatment with genistein affected cell growth and apoptosis-related gene expression via a p53 pathway [13]. In some prostate cancer cell lines, genistein treatment leads to inactivation of the nuclear transcription factor Nuclear Factor-kappa B (NF-κB) via the Akt signaling pathway [14]. Other investigators have shown that the PTEN gene may also reverse chemo-resistance to cisplatin in ovarian cancer through inactivation of the PI3K/Akt cell survival pathway and can be a potential target for the treatment of chemo-resistant cancer [15]. Moreover, genistein also potentiated growth inhibition and apoptosis in certain pancreatic cancer cells by inhibiting Akt and NF-κB [16]. NF-κB is an important regulator of genes involved in cell survival and proliferation; it also plays an important role in the apoptotic pathway [16]. Additionally, tissue transglutaminase, an enzyme involved in protein cross-linking prevents apoptosis induced by cisplatin by activating the NF-κB survival pathway in ovarian tumors [17].

In a recent article it was reported that genistein induces apoptosis in ovarian cancer via different molecular pathways in both wild type and mutated BRCA1 estrogen receptor positive tumors [18]. Genistein also caused cell cycle arrest at G2/M phase in both dose- and time-dependent manner without causing any cytotoxicity [19]. Genistein can also induce both apoptosis and autophagic cell death in ovarian cancer cells [20]; however the role of genistein in chemo-resistant ovarian cancer cells has not been investigated. Therefore, the intent of this study was to evaluate the effect of genistein for sensitization of ovarian cancer cells to conventional cytotoxic chemotherapeutic agents by assessing the effects of combination treatments on cell growth, apoptosis and the DNA binding activity of NF-κB using a paired isogenic cisplatin-sensitive and a cisplatin-resistant ovarian cancer cell line.

## Methods

### Cells, Drugs and Reagents

Paired isogenic cisplatin-sensitive human ovarian cancer cell line A2780 and its cisplatin-resistant clone C200 were received as a generous gift from Dr. Thomas C. Hamilton of Fox Chase Cancer Center, Philadelphia, PA. They were maintained in RPMI media supplemented with 10% fetal bovine serum, insulin and penicillin and streptomycin. C200 cells were grown with cisplatin (3 μM) every 3 passages to maintain resistance. Cells were incubated at 37°C with 5% CO_2_. Taxotere (Aventis Pharmaceuticals, Bridgewater, NJ), was dissolved in DMSO to make a 4 μM stock solution. Cisplatin (Sigma) was dissolved in phosphate buffered saline to make a 1 mM stock solution. Genistein (Toronto Research Chemicals, Inc, ON, Canada) was dissolved in 0.1 M NaHCO_3 _to make a 10 mM stock solution.

### Cell Viability Assay

Paired cells A2780 and C200 were chosen for this study. Cells (2–5 × 10^4^) were seeded in a 96-well culture plate and incubated overnight. Cells were treated with varying concentrations of genistein (5–25 μM), cisplatin (100–2000 nM), taxotere (0.5 – 2 nM) and gemcitabine (10–100 nM) for 48–96 hours. Subsequent experiments were performed with doses that achieved a 40–60% decrease in cell viability in the platinum-sensitive cell line. Cells were pre-treated with genistein for 24 hours followed by combination treatment with genistein and either cisplatin, taxotere or gemcitabine for an additional 48 hours. After 72 hours of total treatment, the cells were incubated at 37°C with 1 mg/mL MTT reagent (Sigma, St. Louis, MO) for 2 hours. The formazan crystals were dissolved in isopropanol. Spectrophotometric absorbance of the samples was determined by the Ultra Multifunctional Microplate Reader (Tecan, Durham, NC USA) at 595 nm. When initial experiments with genistein and cisplatin did not show a significant effect in the resistant cells (C200), the pretreatment interval was increased to 48 hours with genistein alone, followed by 48 hours of combination treatment and the doses of genistein, gemcitabine and taxotere were increased in the C200 cell line.

### Quantification of apoptosis by ELISA

The Cell Death Detection ELISA Kit (Roche, Palo Alto, CA USA) was used for assessing apoptosis in A2780 and C200 cells treated with genistein, cisplatin, taxotere, gemcitabine and their combinations according to the manufacturer's protocol. Briefly, A2780 and C200 cells were treated with 10–25 μM genistein, 250 nM cisplatin, 1–2 nM taxotere and 2–50 nM gemcitabine and the combination of these drugs for 72 to 96 hours. After treatment, the cells were trypsinized and 10,000 cells were added to lysis buffer. The cells were then centrifuged at 20,000 × g for 10 minutes and the supernatant was transferred into anti-histone-coated microtiter plates and incubated at room temperature for 90 minutes. This was followed by anti-DNA-peroxidase incubation for 90 minutes. After unbound antibodies were removed, the nucleosomes were quantified by color development with substrate. The optical densities of the samples were determined by the Ultra Multifunctional Microplate Reader (Tecan, Durham, NC USA) at 405 nm.

### Protein extraction and Western blot analysis

A2780 and C200 cells were seeded in 100 mm dishes and allowed to attach for 24 hours. Cells were then treated with 10–25 μM genistein, 250 nM cisplatin, 1–2 nM taxotere and 2–50 nM gemcitabine and the combination of these drugs for 96 hours to evaluate the effects of treatment on expression levels of survivin, Bcl-2, Bcl-xL, c-IAP1, and was also used for assessing PARP cleavage, an indirect measure of apoptosis. The experiment was carried out for a minimum of three times. Cells were harvested by scraping from culture plates and collecting by centrifugation. Cells were resuspended in lysis buffer consisting of 250 mM NaCl, 50 mM Tris buffer (pH 7.5), 5 mM EDTA, 1% NP40, 0.5% sodium deoxycholate, 0.1% SDS, 50 mM sodium fluoride, 1 mM sodium orthovandate, 1 mM phenylmethylsulfonylfluoride (PMSF), 1 μg/ml pepstatin and a protein inhibitor which contain a broad spectrum of serine, cysteine and metalloproteases (Roche Applied Science, Indianapolis, IN) for 30 minutes on ice. Cell lysates were centrifuged for 20 min. Protein concentration was measured using BCA Protein Assay Kit (Pierce Rockford, IL). The samples were loaded on 7–12% SDS-PAGE for separation and electrophoretically transferred to a nitrocellulose membrane. Each membrane was incubated with monoclonal antibody against Survivin (R & D Systems, Inc. Minneapolis, MN), Bcl-2 (1:200, Calbiochem, San Diego, CA), Bcl-xL, c-IAP1 (Santa Cruz Biotechnology, Santa Cruz, CA), PARP (Biomol, Plymouth, CA), and β-actin (Sigma, St. Louis, MO). Blots were washed with phosphate buffer containing 0.05% Tween (PBST) and incubated with secondary antibodies conjugated with peroxidase. The signal intensity was then measured using chemiluminescent detection system (Pierce Rockford, IL).

### Electrophoretic Mobility Shift Assay (EMSA) for NF-κB activation

EMSA was performed using the Odyssey Infrared Imaging System with NF-κB IRDye labelled oligonucleotide from LI-COR, INC. (Lincoln, NE). The DNA binding reaction included 5 μg of the nuclear extract mixed with oligonucleotide and gel shift binding buffer consisting of (20% glycerol, 5 mM MgCl_2_, 2.5 mM EDTA, 2.5 mM DTT, 250 mM NaCl, 50 mM Tris-HCl pH 7.5, 0.25 mg/ml poly(dI): poly(dC). The reaction was incubated at room temperature in dark for 30 minutes. 2 μl of 10× Orange G loading dye was added to each sample and loaded on the pre-run 8% polyacrylamide gel and ran at 30 mA for 1 hour. NF-κB p65 antibody was used to confirm the super shift and the Rb antibody was used for assessing protein loading control.

### Statistical Methods

Comparisons of survival, and apoptosis between the groups were undertaken by the Student t test. Statistical significance was assumed for a P value of ≤ 0.05.

## Results

### Effects of genistein, cisplatin, gemcitabine and taxotere on the viability of A2780 and C200 ovarian cancer cells

**Figure 1:**
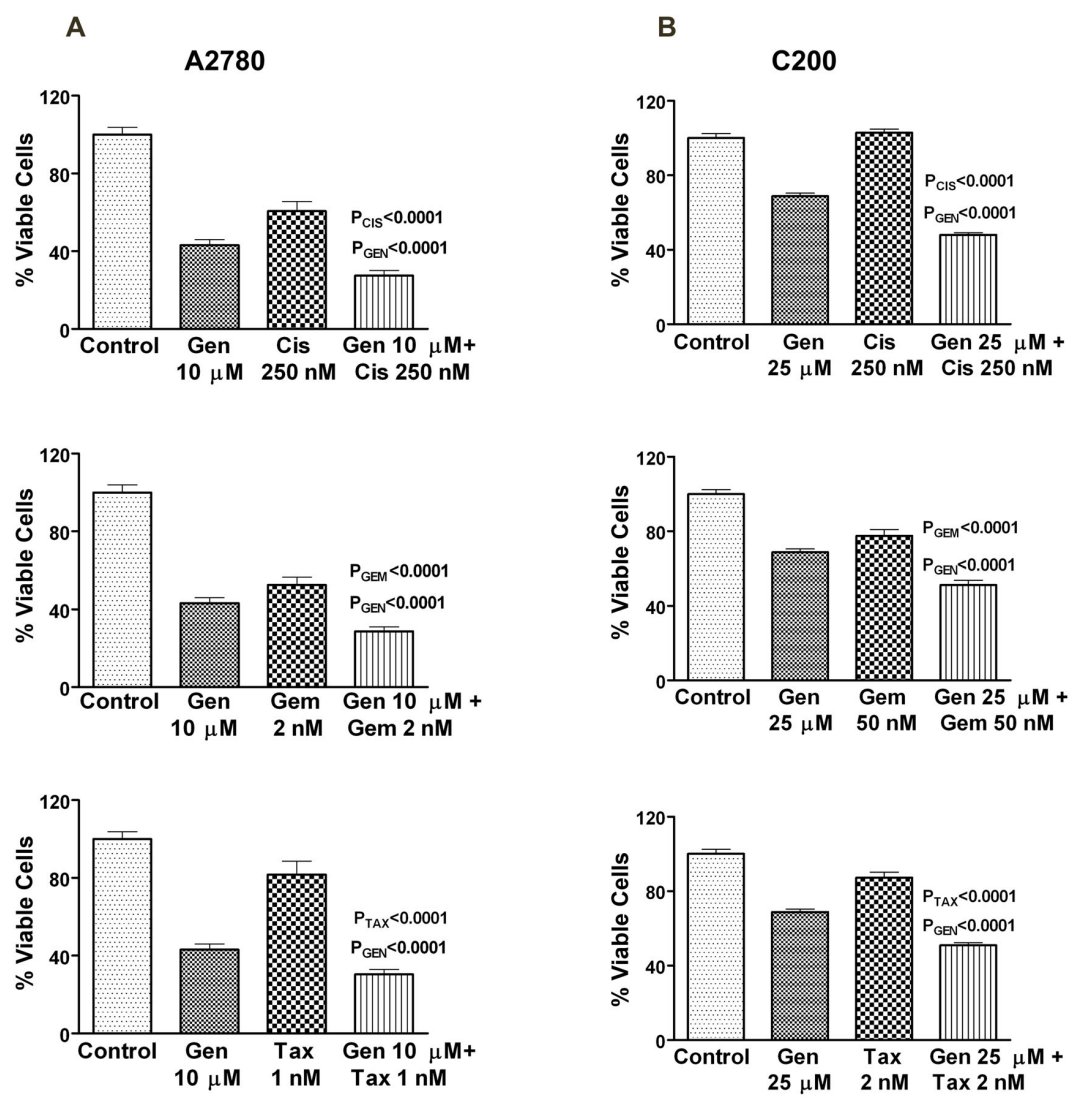
**Growth inhibition of human ovarian cancer cell lines A2780 (A) and C200 (B) treated with genistein (Gen), cisplatin (Cis), gemcitabine (Gem), taxotere (Tax) alone and the combination treatments were evaluated by the MTT assay**. A2780 cells were treated with genistein (10 μM), cisplatin (250 nM), gemcitabine (2 nM) and taxotere (1 nM) and the combination treatment; and the C200 cells were treated with higher doses of genistein (25 μM), gemcitabine (50 nM) and doxetaxel (2 nM) as described under Materials and Methods. There was a significant reduction in the overall cell viability of A2780 and C200 cells treated with the drug combinations compared to cells treated with either drug alone. *P *values shown represent comparisons between each drug alone and the combination of both drugs using t-test.

The viability of A2780 and C200 cells treated with genistein (10–25 μM), cisplatin (250 nM), gemcitabine (2 – 50 nM) and taxotere (1–2 nM) were determined by the MTT assay. The platinum-sensitive A2780 cells were more sensitive to each drug than the platinum-resistant C200 cell line. Pretreatment of the A2780 cells for 24 hours with 10 μM of genistein followed by the combination treatment for 48 hours with each conventional chemotherapeutic agent resulted in a greater and significant inhibition of cell viability compared to each agent alone. On the other hand, C200 cells pretreated with higher than 10 μM genistein concentrations for more than 24 hours (48 hours) followed by combination treatment for additional 48 hours with higher concentration of each conventional chemotherapeutic agent than what was used for A2780 cells also resulted in a significant inhibition of cell viability (Figure 1). Our results showed that genistein can sensitize even the drug-resistant cell line and cause inhibition of cell viability. Further, to assess whether the loss of overall cell viability could also be due to the induction of apoptotic cell death, we examined the effects of genistein, cisplatin, gemcitabine and taxotere, and the combination treatments on apoptotic cell death.

### Induction of apoptosis by genistein, cisplatin, gemcitabine and taxotere in A2780 and C200 ovarian cancer cells

**Figure 2:**
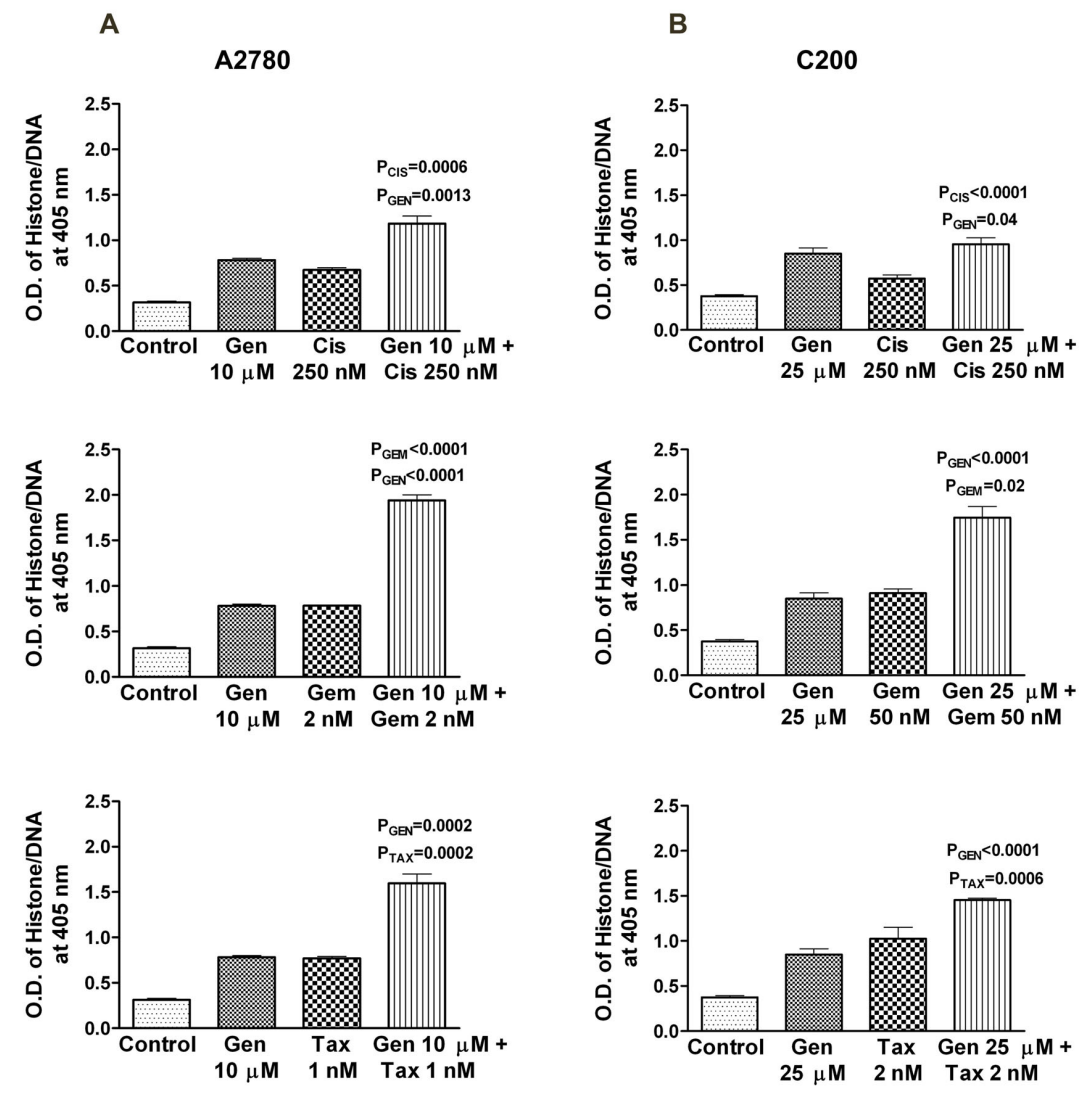
**Induction of apoptosis in human ovarian cancer cell lines A2780 (A) and C200 (B) treated with genistein (Gen), cisplatin (Cis), gemcitabine (Gem) and taxotere (Tax) alone and the combination treatments were evaluated by the ELISA assay**. A2780 cells were treated with genistein (10 μM), cisplatin (250 nM), gemcitabine (2 nM) and taxotere (1 nM) and the combination treatment; and the C200 cells were treated with higher doses of genistein (25 μM), gemcitabine (50 nM) and doxetaxel (2 nM) as described under Materials and Methods. There was a significant potentiation in the induction of apoptosis observed in A2780 and C200 cells treated with both genistein and the other drugs as compared to cells treated with either agent alone.

Apoptosis assays were performed using the A2780 and C200 cell lines to evaluate the mechanism on the inhibition of cell viability using the Cell Death Detection ELISA. For the platinum-sensitive A2780 cell line, 24 hours pretreatment with 10 μM genistein followed by the combination treatment with 250 nM cisplatin, 2 nM gemcitabine and 1 nM taxotere for 48 hours showed a significant increase in apoptosis compared to either drug alone. Increasing the pretreatment interval to 48 hours with 25 μM genistein followed by 48 hours of combination treatment with 250 nM cisplatin, 50 nM gemcitabine and 2 nM taxotere for 48 hours also showed a significant increase in apoptosis compared to either drug alone in resistant cell line (Figure 2). Subsequently, we sought to find further evidence of apoptosis, as presented below.

### The effects of genistein pretreatment and combination treatment on molecules related to apoptosis in A2780 and C200 cells

**Figure 3:**
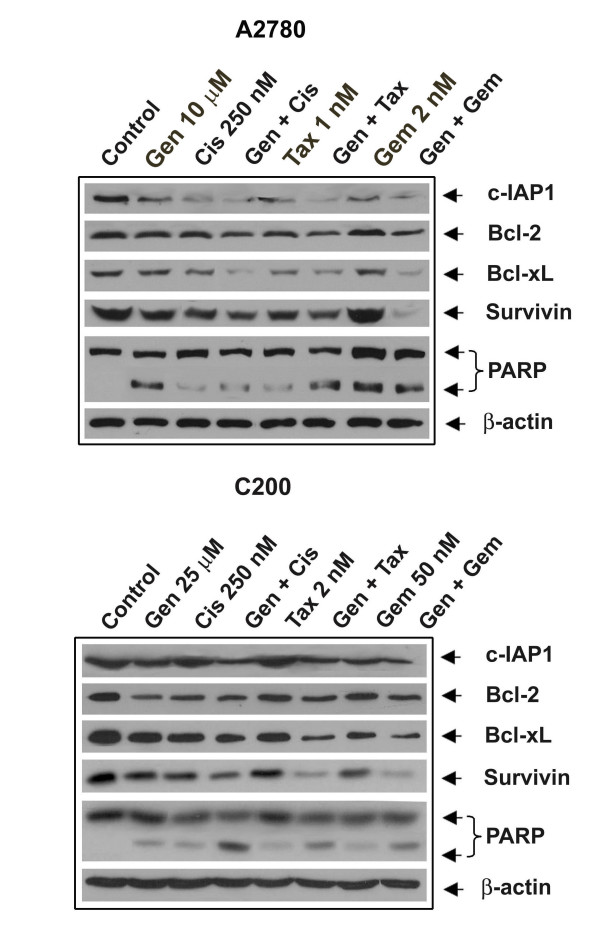
**The expression of c-IAP1, Bcl-2, Bcl-xl, survivin and PARP in A2780 and C200 cells**. Cells untreated or treated with 10 or 25 μM genistein (Gen), 250 nM cisplatin (Cis), the combination (Gen + Cis), 1 or 2 nM taxotere (Tax), the combination (Gen + Tax), 2 or 50 nM gemcitabine (Gem) and the combination (Gen + Gem). β-actin antibodies were used as internal controls for equal loading of proteins. Significant down-regulation of c-IAP1, Bcl-2, Bcl-xl, survivin and PARP was observed in A2780 and C200 cells treated with the combination of genistein and either cisplatin, gemcitabine or taxotere compared to cells treated with either drug alone.

The mechanisms contributing to the potentiation of apoptosis by genistein pretreatment and combination treatment were evaluated in the A2780 and C200 cells. PARP cleavage was determined in A2780 and C200 cells that were treated with genistein (25 μM), cisplatin (250 nM), taxotere (2 nM), gemcitabine (50 nM) alone or the combination treatment of cells pretreated with genistein (Figure 3). We found significantly increased PARP protein cleavage product (85 kDa fragment) after 72 h treatment in A2780 cells (Figure 3). In contrast, C200 cells treated similarly showed comparatively less intense cleaved PARP with combination treatment only. The induction of apoptotic cell death could in part be due to inactivation of important survival genes; and therefore the expressions of pro-survival and anti-apoptotic molecules such as survivin, Bcl-2, Bcl-xL, and c-IAP1, which are transcriptionally regulated by NF-κB, were also evaluated. Expression of Bcl-2, Bcl-xL, survivin, and c-IAP1 proteins were significantly reduced in cells treated with the combination compared to either agent alone in both A2780 and C200 cells. These results suggest that genistein in combination with conventional therapeutics could down-regulate key survival proteins and, in turn, induced apoptotic cell death of both A2780 and C200 cells. Since we found a greater degree of down-regulation of survivin, c-IAP1, Bcl-2, Bcl-xL in cells treated with genistein in combination treatment compared to single agent treatment, we investigated the effect of each treatment on the DNA binding activity of NF-κB.

### Genistein inhibits NF-κB DNA binding activity

**Figure 4:**
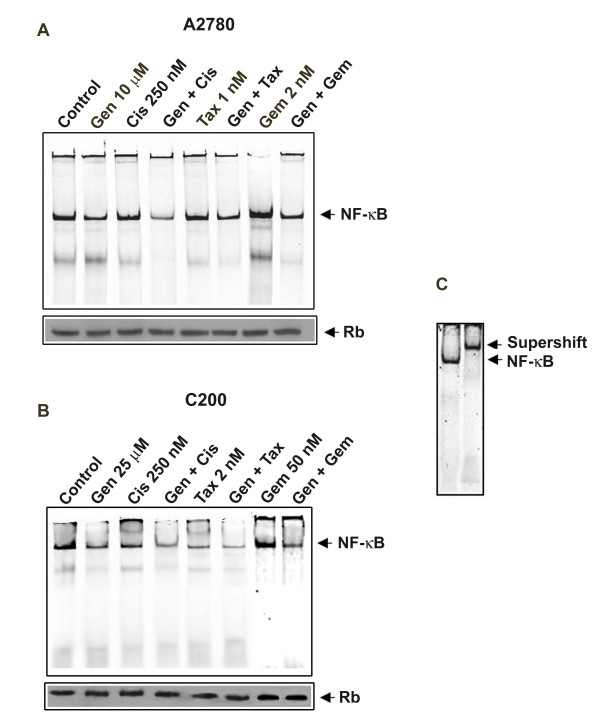
**NF-κB activation in A2780 (A) and C200 (B) human ovarian cancer cells**. Supershift assay (C) showed the formation of bigger complex after addition of anti-NF-κB p65 antibody, resulting in the shift of NF-κB band. Cells untreated or treated with 10 or 25 μM genistein (Gen), 250 nM cisplatin (Cis), the combination (Gen + Cis), 1 or 2 nM taxotere (Tax), the combination (Gen + Tax), 2 or 50 nM gemcitabine (Gem) and the combination (Gen + Gem). Retinoblastoma antibodies were used as internal controls for nuclear protein loading as control. Significant inactivation of NF-κB was observed in A2780 and C200 cells treated with the combination of genistein and either cisplatin, gemcitabine or taxotere compared to cells treated with either drug alone.

Specifically, the effects of pretreatment followed by the combination treatment were studied in the context of NF-κB activation. The treatment of cells with genistein alone significantly down-regulated the DNA binding activity of NF-κB in both the cell lines tested. Interestingly, the combination treatment groups demonstrated greater inhibition of NF-κB compared to the treatment of cells with any of the drugs alone (Figure 4). These results suggest that the pretreatment of cells with genistein sensitized ovarian cancer cells, especially the drug-resistant cells to cisplatin, taxotere, and gemcitabine induced growth inhibition and induction of apoptotic cell death, which is believed to be contributed by the inhibition of survival factors, and inactivation of the DNA binding activity of NF-κB.

## Discussion

Experimental drug resistance to platinum based chemotherapy is a major challenge for the treatment of human ovarian cancer. For ovarian cancer, the standard treatment includes aggressive surgical cytoreduction followed by combination chemotherapy with platinum and a taxane-containing regimen. Although the majority of patients will respond to this therapy, most will recur with chemo-resistant phenotype, which eventually kills patient. Overall, the survival of patients diagnosed with advanced stage disease remains poor, particularly if the tumor is "platinum-resistant" [21-23]. Platinum resistance is defined as tumor progression during initial treatment with a platinum-based combination chemotherapy regimen or recurrence within 6 months of completing front line therapy, and thus is considered intrinsic resistance behavior of ovarian cancer cells. Tumors and metastases may also acquire resistance over time mediated by various mechanisms. Platinum resistance in EOC remains a challenge to effectively treat using currently available second and third line therapies especially in patients having an overall response rate of less than 20%, suggesting that overcoming platinum resistance by novel approaches could be useful for improving the overall survival of patients.

The management of patients diagnosed with ovarian cancer has not changed significantly in the past two decades requiring development of additional treatment protocols for improving overall survival. Emerging evidence suggest that plant-derived non-toxic agents such as curcumin, and genistein show a variety of pharmacological effects [24,25]. Genistein is a naturally occurring isoflavone present in soybeans that has anticancer properties [9,13,16,26]. Therefore, we hypothesized that as in other cancer cell lines; platinum-resistance could be overcome in ovarian cancer cells by combining the conventional chemotherapeutic agents with a non-toxic flavonoid compound, such as genistein.

To test our hypothesis, we sought to assess the efficacy of genistein, a well tolerated naturally occurring substance, in combination with commonly used chemotherapeutic agents in a paired isogenic ovarian cancer cell lines (platinum-sensitive:A2780, platinum-resistant:C200). Though combination therapy was effective in inhibiting cell growth in the platinum-sensitive cell line, genistein pretreatment was required for a response in the platinum-resistant cell line. The observed inhibition of cell growth was subsequently correlated with an increase in the induction of apoptosis. We have further extended our observations to additional cytotoxic conventional chemotherapeutic agents such as taxotere and gemcitabine. We found that genistein pretreatment resulted in the appearance of cleaved PARP under all our experimental conditions, consistent with the increase in apoptosis. This finding suggests that genistein could sensitize ovarian cancer cells to platinum and other conventional chemotherapeutic agents-induced apoptotic cell death and these results are consistent with our previous findings in other cancer cell lines [9,13,16,26].

**Figure 5:**
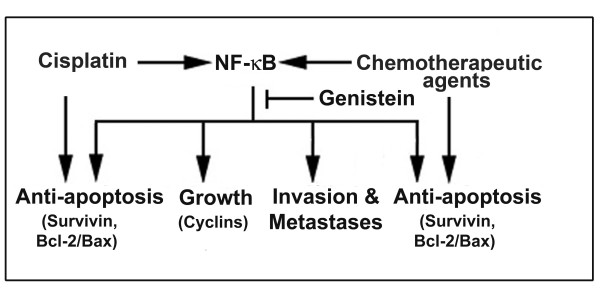
**Schematic diagram of potential mechanism of genistein induced chemo-sensitization of ovarian cancer cells to conventional chemo-therapeutic agents**.

Studies have shown that the underlying resistance to apoptosis is in part due to constitutive activation of NF-κB in pancreatic cancer [27]. Our results strongly suggest that the resistance of ovarian cancer cells treated with cisplatin could in part be also due to the activation of NF-κB and that the chemo-sensitization could be due to genistein-induced inactivation of NF-κB signaling, resulting in the inhibition of cell proliferation and induction of apoptosis (Figure. 5). Our hypothesis is also supported by the evidence of down-regulation of the important anti-apoptotic proteins such as Bcl-2, Bcl-xL. survivin and c-IAP2, which also happens to be the downstream genes of NF-κB. C-IAP-2 and survivin are members of the anti-apoptotic IAP (Inhibitors of Apoptosis) family of proteins suppressing apoptosis and their expression in tumors has been associated with poor prognosis and increased tumor recurrence in many tumors. Our findings reveal the expression of these anti-apoptotic proteins is decreased by genistein, and is probably driven by NF-κB activation suggesting another possibility for inhibiting tumor and that NK-κB, survivin and IAP'S may make an important contribution to the development of chemo-resistance.

Multiple pathways linked with cisplatin resistance have been reported by many researchers. A synthetic triterpenoid inhibited IL-6-Stat-3 pathway which is one of the key pathway contributing to drug resistance in ovarian cancer [3]. Pretreatment of cisplatin resistant ovarian cancer cells with trichostatin, a histone deacetylase inhibitor overcomes mitochondria-dependent apoptosis by restoring p73 and Bax expression [22]. A small molecule inhibitor triethylenetetramine inhibited superoxide dismutase 1 which is over expressed in cisplatin resistant cells by enhancing the cisplatin sensitivity in the resistant cells [21]. These findings appear to lend support for our current observation and as such consistent with the role of genistein in the prevention of cisplatin-induced renal injury in mice [28], and its biological effects on breast [13], pancreas [27,29], and melanoma cells [30], associated with the inhibition in the translocation of p65 subunit of NF-κB in the nucleus, which is otherwise increased by the cisplatin treatment.

## Conclusion

In conclusion, the evidence provided by this study lend strong support for our hypothesis that genistein pre-treatment could overcome drug-resistance in ovarian cancer cells as documented by increased cell growth inhibition and the induction of apoptotic cell death. We have also shown that the genistein mediated chemo-sensitization of ovarian cancer cells to conventional chemotherapeutic agents was partly due to inactivation of the DNA binding activity of NF-κB and its downstream genes. Our results warrant further pre-clinical and clinical studies for assessing the value of genistein in overcoming drug-resistance in ovarian cancer in order to improve the overall survival of patients diagnosed with ovarian cancer especially those with drug-resistant characteristics.

## Competing interests

The authors declare that they have no competing interests.

## Authors' contributions

LAS and SA collected data for the study and prepared the original manuscripts. SB carried out the supershift assay; ARM and RTM supervised the project. FHS directed the research. All authors approved the final manuscript.
